# ﻿*Pseudobaorangia* (Boletaceae, Boletales), a new genus for *Boletuslakhanpalii*

**DOI:** 10.3897/mycokeys.119.144869

**Published:** 2025-07-10

**Authors:** Fan Zhou, Junbo Zhang, Shaoxiong Liu, Qimeng Liu, Xi Luo, Xiaokun Luo, Lei Wang, Chunli Liu, Xiang Guo, Yunli Feng, Dafeng Sun, Rong Hua

**Affiliations:** 1 Kunming Edible Fungi Institute of All-China Federation of Supply and Marketing Cooperatives, Yunnan 650221, China Kunming Edible Fungi Institute of All-China Federation of Supply and Marketing Cooperatives Kunming China; 2 Yunnan Academy of Edible Fungi Industry Development, Yunnan 650221, China Yunnan Academy of Edible Fungi Industry Development Kunming China

**Keywords:** *Baorangia*, Boletaceae, new genus, *Pseudobaorangia*, taxonomy

## Abstract

Bolete specimens collected in Baima Snow Mountain Nature Reserve, Yunnan Province, were identified as *Pseudobaorangialakhanpalii*, based on morphological and phylogenetic analyses. The ITS sequences of our collections exhibited a high similarity with *Boletuslakhanpalii* (99.53% for KEF13271 and 99.44% for KEF13272). However, because of limited molecular data and complex taxonomic relationships of Boletaceae, the phylogenetic placement of *B.lakhanpalii* could not be clearly determined. This study conducted a phylogenetic analysis by combining nrLSU, *RPB2* and *TEF1-α* genes, and the results showed that this species has a close phylogenetic relationship with *Baorangia*, sharing some morphological characteristics, but also having several highlighting differences. Therefore, a new genus *Pseudobaorangia* is proposed with *P.lakhanpalii* as the type species and its detailed description is provided.

## ﻿Introduction

*Boletuslakhanpalii* was originally described by Das et al. (2015). However, because of insufficient molecular data, its taxonomic relationship is still questionable. Initial ITS sequence analyses revealed a close phylogenetic relationship with *B.bicolor* Peck, *B.fragrans* Vittad. and *Xerocomellusarmeniacus* (Quél.) Šutara. Notably, current taxonomic revisions (Index Fungorum, accessed 2025) have reclassified these taxa as *Baorangiabicolor* (Kuntze) G. Wu, Halling & Zhu L. Yang (Wu et al. 2015), *Lanmaoafragrans* (Vittad.) Vizzini, Gelardi & Simonini (Crous et al. 2018) and *Rheubarbariboletusarmeniacus* (Quél.) Vizzini, Simonini & Gelardi (Frank et al. 2020), respectively. Therefore, the taxonomic position of *B.lakhanpalii* remained undetermined.

*Baorangia* was established by Wu et al. (2015) and characterised by a very thin hymenophore (pileus context thickness 3–5 times that of the hymenophore), light yellow to yellow context slowly turning blue when injured, hymenophore quickly turning pale green to blue-green when bruised, a smooth stipe with occasional reticulum confined to the apex and mostly found in mixed forests under trees of the Fagaceae and Pinaceae. Basidiospores smooth, pale yellow to brownish-yellow and subfusiform to elongated subfusiform. Until now, seven species have been described in this genus (Wu et al. 2015; Crous et al. 2018; Phookamsak et al. 2019; Zhang et al. 2021).

The ITS sequences of the species which we gathered showed 99.53% (KEF13271, 639 bp) and 99.44% (KEF13272, 539 bp) similarity to *B.lakhanpalii*. Combined morphological studies infers that the newly-collected Chinese specimens are to be assigned to *B.lakhanpalii*. Considering that Das et al. (2015) failed to clarify the taxonomic placement of *B.lakhanpalii* in their previous study, the combined nrLSU, *RPB2* and *TEF1-α* gene fragments were used in this study to define the phylogenetic relationship of this species. The results are presented and discussed herein.

## ﻿Materials and methods

### ﻿Specimen collection and preservation

All samples were gathered in Yunnan Province, China and the morphological features of the basidiomata documented and photographed on-site. Fresh basidiomata were dried at 41–56 °C for 15 hours by a food-grade fruit dryer, before being deposited in the Specimen Museum of the Kunming Edible Fungi Research Institute (**KEF**).

### ﻿Morphological studies

Macroscopic features of basidiomata came from records and photographs. Colour codes were based on “Taschenlexikon der Farben 3” (Kornerup and Wanscher 1981). Delineation of microstructure was obtained from rehydration of desiccated samples in aqueous solution, followed by staining with Congo red. Observation and description of microstructures were based on previous studies (Li et al. 2011; Zeng et al. 2012; Hosen et al. 2013; Zeng et al. 2014; Zhou et al. 2022, 2023). Microstructures of rehydrated sections were observed with a LEICA DM5000 B microscope (Leica GmbH, Germany) and drawn by hand.

The numerical notation n/m/p represents the examination of n basidiospores derived from m basidiomata across p collections. The dimensions of the basidiospores are represented in the standardised format “(a) b-c (d)”, where the range b-c represents a minimum of 90% of the measured values, while extremes (a) and (d) denote exceptional measurements beyond this primary distribution. The ratio of basidiospore length to width is denoted as *Q*, *Qm* representing the average *Q* of basidiospores ± sample standard deviation. (Zeng et al. 2018; Zhou et al. 2023).

### ﻿DNA extraction, PCR amplification and sequencing

DNA was successfully extracted from the exsiccate specimens using an optimised CTAB procedure (Pahlich and Gerlitz 1980; Huang et al. 2000). Primer sets ITS1-ITS4 (White 1990), LR0R-LR5 (Vilgalys and González 1990; James et al. 2006), fRPB2-5F-fRPB2-7cR (Liu et al. 1999) and TEF1-983F-TEF1-1567R (Rehner and Buckley 2005; Li et al. 2014) were applied to amplify the internal transcribed spacer region (ITS), the large ribosomal subunit sequence region (nrLSU), the RNA polymerase II second largest subunit gene (*RPB2*) and the translation elongation factor 1-α gene (*TEF1-α*), respectively. The PCR reaction conditions followed Wu et al. (2014) and Zhou et al. (2022, 2023). The PCR products were dispatched to TSINGKE Biological Technology (Yunnan, China) for sequencing.

### ﻿Phylogenetic analyses

Sequences were visualised and modified using MEGA X (Kumar et al. 2018) and BLASTed against GenBank database for homology search. The ITS sequences of our collections exhibited the highest similarity to *Boletuslakhanpalii* (99.53% with KEF13271, 639 bp and 99.44% with KEF13272, 539 bp). However, *B.lakhanpalii* only had two ITS sequence in GenBank and the BLAST results for nrLSU, *RPB2* and *TEF1-α* sequences showed significant differences, making it difficult to determine its phylogenetic relationship. The data of similar genera were downloaded, based on the BLAST results of nrLSU, *RPB2* and *TEF1-α* sequences of KEF13271 and KEF13272, and all available data are listed in Table 1. ITS sequences were not suitable for inferring phylogenetic relationships between different genera in Boletaceae (Wu et al. 2023); therefore, ITS sequences were excluded from the final multi-gene analysis, but are provided in the taxonomy section.

**Table 1.** The species gene fragments and their voucher, locations and GenBank accession numbers used in this study. Newly-generated sequences are marked in bold.

Species	Voucher	Locality	nrLSU	*RPB2*	*TEF1-α*	Reference
*Baorangiaalexandri*	LE 254265	Russia	MH036170	—	—	(Crous et al. 2018)
*Baorangiaalexandri*	LE 254266	Russia	MH036169	—	—	(Crous et al. 2018)
*Baorangiabicolor*	Mushroom Observer #282994	USA	—	—	MH347322	GenBank
*Baorangiaduplicatopora*	FHMU5877	China	MW473483	—	MW485968	(Zhang et al. 2021)
*Baorangiaduplicatopora*	FHMU5878	China	MW473482	—	MW485967	(Zhang et al. 2021)
*Baorangiaemileorum*	TO HG171015	Italy	MH036173	MG897443	MG897433	(Phookamsak et al. 2019)
*Baorangiaemileorum*	GS 10213	Italy	MH036171	KM605179	KJ184570	(Wu et al. 2015)
*Baorangiamajor*	OR404	Thailand	—	—	MG897432	GenBank
*Baorangiamajor*	OR0486	Thailand	—	MG897443	MG897433	GenBank
*Baorangiapseudocalopus*	HKAS 75739	China	KJ184558	KM605179	KJ184570	(Wu et al. 2015)
*Baorangiarufomaculata*	BOTH4144	Thailand	KF030248	MG897435	MG897425	(Nuhn et al. 2013; Phookamsak et al. 2019)
*Boletusbotryoides*	HKAS53403	China	—	KT990375	KT990738	(Wu et al. 2016)
*Butyriboletusbrunneoides*	BJTC FM1816	China	NG_149026	OL771223	OL799255	(Fu et al. 2022)
*Butyriboletusregius*	KUN-HKAS 84878	China	MT264910	MT269661	MT269659	(Wu et al. 2020)
*Butyriboletussinoregius*	BJTC FM755	China	OL721748	OL771224	OL799252	(Fu et al. 2022)
*Caloboletusgriseoflavus*	BJTC FM2438	China	OR655183	OR659935	OR659984	(Mao et al. 2023)
*Caloboletusguanyui*	FHMU2224	China	MH879707	MH879750	MH879735	(Chai et al. 2019)
*Caloboletusyunnanensis*	HKAS69214	China	KJ184556	KT990396	KJ184568	(Wu et al. 2016; Zhao et al. 2014a)
*Hongoboletusventricosus*	TNS-F-44611	Japan	OQ888732	OQ873507	—	(Wu et al. 2023)
*Hongoboletusventricosus*	TNS-F-44612	Japan	OQ888733	OQ873508	—	(Wu et al. 2023)
*Hongoboletusventricosus*	HKAS 63598	Japan	KF112317	KF112663	KF112152	(Wu et al. 2014)
*Imleriafloridana*	Franck_4235	USA	MN584689	MN584683	MN584686	(Farid et al. 2020)
*Lanmaoamacrocarpa*	FHMU2212	China	MH879685	—	MH879714	(Chai et al. 2019)
*Lanmaoapseudosensibilis*	DS615-07	USA	KF030257	—	KF030407	(Nuhn et al. 2013)
*Lanmaoasublurida*	Farid_631	USA	MW662578	MW737464	MW737487	(Farid et al. 2021)
*Neoboletusbrunneorubrocarpus*	HKAS:76660	China	KF112328	KF112731	KF112180	(Farid et al. 2021)
*Neoboletuserythropus*	VDKO0690	Belgium	—	KT824015	KT824048	(Raspé et al. 2016)
*Neoboletusluridiformis*	AT2001087	UK	JQ326995	—	JQ327023	(Halling et al. 2012)
*Neoboletusobscureumbrinus*	FHMU2055	China	MH879695	MH879743	MH879724	(Chai et al. 2019)
***Pseudobaorangialakhanpalii***	**KEF13271**	China	**PQ773310**	**PQ824861**	**PQ824859**	**This study**
***Pseudobaorangialakhanpalii***	**KEF13272**	China	**PQ773311**	—	**PQ824860**	**This study**
Retiboletusaff.griseus	HKAS59460	China	JQ928626	JQ928601	JQ928580	(Hosen et al. 2013)
*Retiboletusater*	Li1215	China	MT010611	ON004079	MT010621	(Li et al. 2022; Liu et al. 2020)
*Retiboletusfuscus*	HKAS74756	China	KT990636	KT990467	KT990830	(Wu et al. 2016)
*Retiboletuskauffmanii*	HKAS63584	China	KT990634	KT990465	KT990828	(Wu et al. 2016)
*Rubroboletusesculentus*	K. Zhao893	China	KY272129	KY272135	KY272138	(Zhao and Shao 2017)
*Rubroboletuslatisporus*	HKAS 80358	China	NG_059540	KP055029	KP055020	(Zhao et al. 2014b)
*Rubroboletusserpentiformis*	HKAS126557	China	OQ888723	OQ873498	OQ873460	(Wu et al. 2023)
*Suillellusluridus*	VDKO0241b	Belgium	—	KT824014	KT824047	(Raspé et al. 2016)
*Suillellusolivaceus*	BJTC FM1755	China	OR655212	OR659964	OR660011	(Mao et al. 2023)
*Suillellusyunnanensis*	HKAS126548	China	OQ888730	OQ873505	OQ873467	(Wu et al. 2023)
*Xerocomellusinflatus*	W3272	China	OR704326	OR573955	OR663937	(Wang et al. 2024)
*Xerocomellustenuis*	MHKMU R. Xue 100	China	PP179418	PP195256	PP230529	(Xue et al. 2024)
*Xerocomusnothofagi*	JAC12297	New Zealand	OP141516	—	—	GenBank
*Xerocomusrufostipitatus*	PDD 101779	New Zealand	OP141589	—	—	GenBank

All single-gene datasets were separately submitted to MAFFT version 7 (Katoh and Standley 2013) online platform for alignment. The *RPB2* and *TEF1-α* introns could be well aligned and retained in the final analyses (Wu et al. 2014). The single-gene phylogenetic trees showed no conflict in topology and the corresponding alignments were then concatenated in PhyloSuite (Zhang et al. 2020). Maximum Likelihood (ML) and Bayesian Inference (BI) analyses were performed on the concatenated dataset using RAxML v. 8 (Stamatakis 2014) and MrBayes v. 3.2 integrated into PhyloSuite (Ronquist et al. 2012; Zhang et al. 2020). PartitionFinder 2 (Lanfear et al. 2016) was used to evaluate the best-fit models for the combined dataset (nrLSU + *RPB2* + *TEF1-α*, for a total of 2,395 bp) using the Bayesian Information Criterion (BIC), where character sets were nrLSU = 1–926, *RPB2* = 927–1692 and *TEF1-α* = 1693–2395. In RAxML v. 8, the GTRGAMMA + I model was used, in accordance with the evaluation results. One thousand repetitions were set for non-parametric bootstrap analysis (Felsenstein 1985). In BI analyses, the best-fit substitution model was estimated for each character set in the dataset using PartitionFinder (Lanfear et al. 2016). The Markov Chain Monte Carlo (MCMC) parameters were set as follows: 2,000,000 generations, sampling every 1,000^th^ generation, two runs of four chains each and the posterior probabilities were calculated after discarding the first 25% generations as burn-in. Phylogenetic trees were visualised using FigTree version 1.4.4. Only the values of BS ≥ 50% and PP ≥ 0.9 are displayed in the phylogenetic tree.

## ﻿Results

### ﻿Molecular data

**Figure 1.**
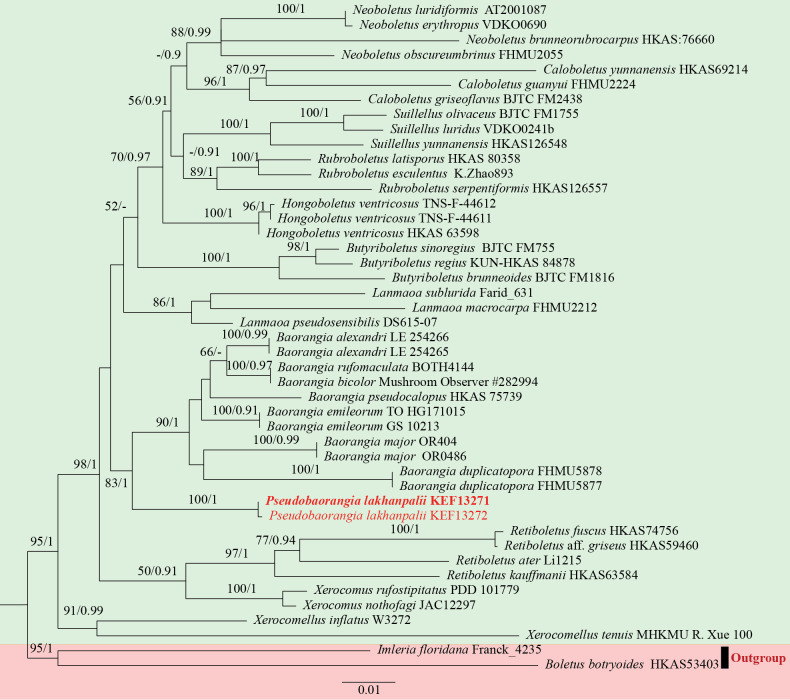
Maximum Likelihood phylogenetic tree of *Pseudobaorangia* and related genera, based on the combined nuclear dataset (nrLSU + *RPB2* + *TEF1-α*). BS ≥ 50% and PP ≥ 0.9 are shown above supported branches.

Seven novel sequences, two each for ITS, nrLSU and *TEF1-α* and one for *RPB2*, were generated from two collections (KEF13271, KEF13272). The best-fit substitution models were SYM + I + G for the nrLSU character set and K80 + G for the *RPB2* and *TEF1-α* character sets. After 2,000,000 generations, the average deviation of splitting frequency was 0.007066. The concatenated dataset (nrLSU + *RPB2* + *TEF1-α*) contains 45 operating taxonomic units (OTUs) with 2395 bases, the nrLSU dataset contained 39 sequences and 926 characters, the *RPB2* and *TEF1-α* datasets consist of 33 accessions with 766 nucleic acids and 39 taxonomic units with 703 bases, respectively. The ML tree and BI tree share the same topology and the data have been submitted to Dryad (https://doi.org/10.5061/dryad.w3r228134); Consequently, only ML trees annotated with both bootstrap support (BS) and posterior probability (PP) values are shown in Fig. 1.

### ﻿Taxonomy

#### ﻿ Pseudobaorangia

Taxon classificationFungiBoletalesBoletaceae

D.F. Sun, R. Hua, F. Zhou & J.B. Zhang gen. nov.

168102BA-70AA-5362-BD86-A91B33D08E00

Fungal Names: FN 571906

##### Etymology.

The epithet “*Pseudobaorangia*” indicates a close phylogenetic relationship with *Baorangia*.

##### Diagnosis.

***Basidiomata*** xerocomoid, with tubular hymenophore. ***Pileus*** hemispherical to convex when young, applanate to depressed when mature; surface dry, tomentose, cracking into scales with age, pale yellow brown; ***margin*** enrolled when young; ***context*** white to pale yellowish-white, quickly turning blue when injured. ***Hymenophore*** slightly depressed to decurrent at stipe apex; ***pores*** compound, labyrinthoid when young then angular to irregular when mature, quickly changing blue when bruised; ***tubes*** orange-yellow to yellow-brown, turning blue when damaged. ***Stipe*** pruinose when young, smooth with spots in age, clavate, solid, greyish-brown to dark red; ***context*** orange-yellow to reddish-brown to greyish brown from the apex down to the base, unchanging when cut; ***basal mycelium*** yellow to pale grey. ***Basidiospores*** smooth, thin walled, subellipsoid to amygdaloid, greyish-yellow in water; ***cheilocystidia*** uncommon, subclavate to fusiform with blunt apex. ***Pleurocystidia*** uncommon, clavate to subfusiform or fusiform with slender apex.

##### Type species.

*Boletuslakhanpalii* K. Das, D. Chakraborty, A. Baghela, S.K. Singh & B.T. M. Dentinger.

#### ﻿ Pseudobaorangia lakhanpalii

Taxon classificationFungiBoletalesBoletaceae

(K. Das, D. Chakraborty, A. Baghela, S.K. Singh & B.T. M. Dentinger,) D.F. Sun, R. Hua, F. Zhou & J.B. Zhang comb. nov.

51BB53A4-1281-5559-8402-FF158F7FD06A

Fungal Names: FN 571907

Figs 2 , 3

##### Basionym.

*Boletuslakhanpalii* K. Das, D. Chakraborty, A. Baghela, S.K. Singh & B.T. M. Dentinger, Sydowia 67: 14, 2015.

##### Description.

***Pileus*** 3–5 cm in diameter, greyish-brown (7E3) to deep yellow brown (5E3), hemispherical to convex when young, applanate to depressed at maturity, surface dry, with a pale yellowish-brown (5C3) tomentum and pale yellowish-white (4A2) margin slightly enrolled when young, tending to crack into scales with age; ***context*** up to 9 mm at the centre of pileus when mature, white (1A1) to light yellowish-white (2A2), quickly turning blue when cut. ***Hymenophore*** slightly depressed and decurrent at stipe apex; ***pores*** labyrinthine when young, angular to irregularly arranged when mature, 1–2 per mm, light orange-yellow (4A3) to orange-yellow (4A5), quickly bluing when touched; ***tubes*** 3–6 mm long (in age), orange-yellow (4A3) to yellow-brown (4D3), turning blue when damaged. ***Stipe*** 3–5 × 0.5–1.2 cm, clavate, central, solid, surface pruinose or tiny dots, greyish-brown (1F4) to dark red (9E3); ***context*** is orange-yellow (5B3) to reddish-brown (8D3) to greyish-brown (8E2) from apex to the base, no reaction when cut; ***basal mycelium*** yellow (5A2) to pale grey (5B1). ***Odour*** indistinct.

**Figure 2.**
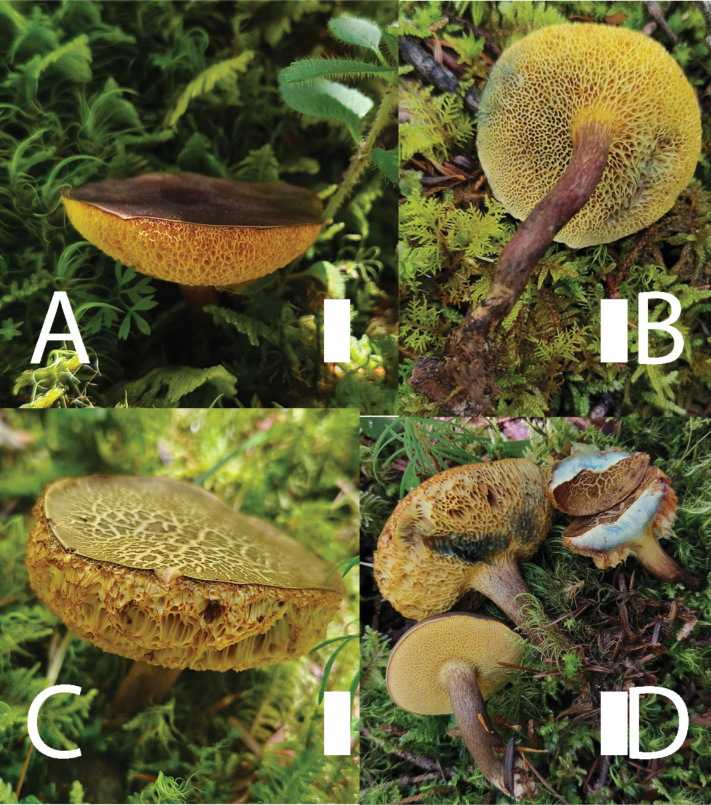
Basidiomata of *Pseudobaorangialakhanpalii*. **A, B.** KEF13271; **C, D.** KEF13272. Scale bars: 1 cm. Photos by F. Zhou.

***Basidia*** four-spored, 32.7–48 × 9.8–12.7 μm, clavate, thin-walled, hyaline to pale grey in water, sterigmata 3–6.6 μm long. ***Basidiospores*** [100/3/2] (8.8–)9.3–12.3(–13.3) × (4.1–)4.5–5.5(–6) μm, *Q* = (1.6–)1.7–2.5(–2.7), *Qm* = 2.17 ± 0.22, smooth, thin-walled, subellipsoid to amygdaliform, greyish-yellow in water. ***Hymenophoral trama*** composed of 4.6–9.5 μm wide, slightly thick-walled interwoven hyphae, hyaline to pale grey in water. ***Cheilocystidia*** 27.1–46.4 × 8.4–10.7 μm, uncommon, thin-walled, smooth, subclavate to fusiform with blunt apex, hyaline to pale grey in water. ***Pleurocystidia*** 38.8–62.5 × 8.5–9.8 μm, uncommon, thin-walled, smooth, clavate to subfusiform or fusiform with slender apex, hyaline to pale grey in water. ***Pileipellis*** a trichoderm composed of interwoven thin-walled hyphae with a diameter of 3.5–6.3 μm, 66–135 μm thick, hyaline to greyish-brown in water; ***terminal cells*** clavate or subcylindrical with blunt apex, 10.3–24.9 × 3.6–5.7 μm. ***Pileus trama*** composed of 3.5–7.6 μm wide, hyaline to pale grey, thin-walled hyphae. ***Stipitipellis*** 40–86 μm thick, clavate, ventricose and subcylindrical terminal cells (19–45 × 4.5–9.5 μm) with rounded apex, hyaline to greyish-brown to yellowish-brown hyphae in water; ***stipe trama*** composed of 2.5–6 µm wide, parallel and hyaline hyphae. ***Clamp connections*** absent.

**Figure 3.**
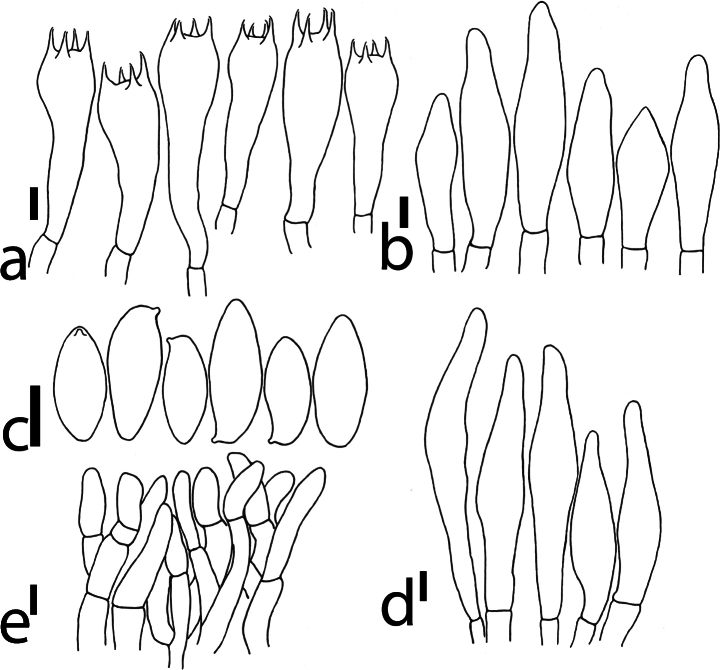
Microstructure of *Pseudobaorangialakhanpalii* (KEF13271). **a.** Basidia; **b.** Cheilocystidia; **c.** Basidiospores; **d.** Pleurocystidia; **e.** Pileipellis. Scale bars: 6 μm. Drawings by F. Zhou.

##### Distribution.

Only found in Yunnan Province, China and Sikkim, India.

##### Habitat.

Mixed forest of *Abiesgeorgei* and *Quercusguyavifolia*.

##### Specimens examined.

China • Yunnan Province: Diqing Tibetan Autonomous Prefecture, Deqin County, Shengping Town, Quzonggong, Baima Snow Mountain Nature Reserve; 13 August 2024; 28°19.64'N, 99°3.32'E; elev. 3830 m; F. Zhou; 813-75 (KEF13271); • same location; 13 August 2024; 28°19.63'N, 99°3.34'E; elev. 3830 m; F. Zhou; 813-78 (KEF13272).

##### GenBank accession numbers.

ITS = PQ773312, nrLSU = PQ773310, *RPB2* = PQ824861, *TEF1-α* = PQ824859 (KEF13271); ITS = PQ773313, nrLSU = PQ773311, TEF*1-α* = PQ824860 (KEF13272).

## ﻿Discussion

Although Das et al. (2015) suggested the possibility of *Boletuslakhanpalii* as an independent new genus and highlighted its differences from *Boletus*, *B.lakhanpalii* was ultimately classified under *Boletus* because of the unclear phylogenetic relationships amongst different clades of Boletaceae at that time. With the continuous development of Boletaceae taxonomy, additional genes were used, resulting in an increasing number of sequences and new taxa (Wu et al. 2015, 2016, 2023; Vadthanarat et al. 2018; Chai et al. 2019; Zhang et al. 2019; Frank et al. 2020; Farid et al. 2021; Li and Yang 2021; Das et al. 2023; Vadthanarat et al. 2024; Wang et al. 2024). Therefore, the phylogenetic relationships of Boletaceae species have become gradually clearer.

In the phylogenetic analysis, the specimens we collected formed an independent lineage with the highest support (BS = 100%, PP = 1) and clusters as a sister clade to *Baorangia* with good statistical support (BS = 83%, PP = 1).

In a morphological comparison, *Baorangia* can be distinguished by its: 1) thin hymenophore (context in the middle part of the pileus is 3–5 times thicker than that of the hymenophore); 2) a light yellow to yellow stipe, its context turning pale blue when cut; 3) much larger and fleshy basidiomes with a boletoid habit; 4) simple, small and roundish pores (with the only exception of *B.major* Raspé & Vadthanarat, (Phookamsak et al. 2019)) and 5) broader geographical distribution across the Northern Hemisphere, while *P.lakhanpalii* appears to be restricted to subalpine regions of the Himalayan range (Wu et al. 2015; Crous et al. 2018; Phookamsak et al. 2019; Zhang et al. 2021).

It also shares similar characteristics with *Xerocomus* s. str. (*X.subsplindidus*, *X.subparvus* and *X.yunnanensis*) and *Xerocomellusdiffractus*. However, *Xerocomussubsplindidus* has a white context and does not change when injured, longer basidiospores (9–15.5(–16) × 4–5.5(–6) µm) and cheilocystidia (39–65 × 8.5–12 µm) and thicker pleurocystidia (31–82 × 10–13 µm) (Zhou et al. 2023). *Xerocomussubparvus* has thinner stipe (2.5–5 × 0.2–0.7 cm) and basidiospores ((8.5) 9–10.5 (11.5) × (3) 3.5–4 (4.5) µm) and larger pleuro-and cheilocystidia (42–90 × 10–20 µm) and terminal cells of pileipellis 28–70 × 10–18 µm (Wu et al. 2016). *Xerocomusyunnanensis* can be differentiated by the fresh yellow tubes staining red-brown slowly when young and white to yellowish context of stipe, changing bluish indistinctly when cut and larger pleuro-and cheilocystidia (35–85 × 10–20 µm) and the terminal cells of pileipellis 30–50 × 7.5–15 µm (Wu et al. 2016). *Xerocomellusdiffractus* has larger pileus (3–10(–14) cm) and stipe (4–10 × 0.8–2 cm) and longer basidiospores ((12.5–)12.9–16.2 × (4.7–)5.1–6.2 µm) and shorter basidia (18.5–27.2 × 7.5–11.5 µm).

Based on the above information, *Pseudobaorangia* is established as a new genus, with *P.lakhanpalii* as the type species.

## Supplementary Material

XML Treatment for Pseudobaorangia

XML Treatment for Pseudobaorangia lakhanpalii

## ﻿Additional information

### Author ORCIDs

Fan Zhou https://orcid.org/0000-0001-9927-3073

Shaoxiong Liu https://orcid.org/0009-0002-2115-9849

Qimeng Liu https://orcid.org/0009-0008-4061-6279

Xi Luo https://orcid.org/0000-0001-7112-1895

Yunli Feng https://orcid.org/0009-0000-3370-5996

Dafeng Sun https://orcid.org/0000-0002-2303-484X

Rong Hua https://orcid.org/0009-0003-3261-4912

### Data availability

All of the data that support the findings of this study are available in the main text.
